# A Novel Strategy for Gene Selection of Microarray Data Based on Gene-to-Class Sensitivity Information

**DOI:** 10.1371/journal.pone.0097530

**Published:** 2014-05-20

**Authors:** Fei Han, Wei Sun, Qing-Hua Ling

**Affiliations:** 1 School of Computer Science and Communication Engineering, Jiangsu University, Zhenjiang, China; 2 School of Computer Science and Engineering, Jiangsu University of Science and Technology, Zhenjiang, China; Technische Universität Dresden, Medical Faculty, Germany

## Abstract

To obtain predictive genes with lower redundancy and better interpretability, a hybrid gene selection method encoding prior information is proposed in this paper. To begin with, the prior information referred to as gene-to-class sensitivity (GCS) of all genes from microarray data is exploited by a single hidden layered feedforward neural network (SLFN). Then, to select more representative and lower redundant genes, all genes are grouped into some clusters by K-means method, and some low sensitive genes are filtered out according to their GCS values. Finally, a modified binary particle swarm optimization (BPSO) encoding the GCS information is proposed to perform further gene selection from the remainder genes. For considering the GCS information, the proposed method selects those genes highly correlated to sample classes. Thus, the low redundant gene subsets obtained by the proposed method also contribute to improve classification accuracy on microarray data. The experiments results on some open microarray data verify the effectiveness and efficiency of the proposed approach.

## Introduction

One of the major applications of microarray data analysis is to perform sample classification between different disease phenotypes, for diagnostic and prognostic purposes [1]–[5]. The classification involves a wide range of algorithms such as differential gene expression analyses, clustering analyses and supervised learning [6]. Gene selection is one of the critical steps in the course of the classification of microarray data [7], [8]. Selecting a useful gene subset not only decreases the computational complexity, but also increases the classification accuracy.

The methods for gene selection are broadly divided into three categories: filter, wrapper and embedded methods [9]. A filter method relies on general characteristics of the training data to select genes without involving any classifier for evaluation. Most filter methods consider each feature separately with ignoring feature dependencies, which may lead to worse classification performance when compared to other types of feature selection methods [10]. In addition to considering feature dependencies, wrapper methods take into account the interaction between feature subset search and model selection. However, wrapper methods have a higher risk of overfitting than filter ones and are very computationally intensive [11]. Embedded methods have the advantage that they include the interaction with the classification model, while being far less computationally intensive than wrapper methods [12].

In recent years, many methods combined with population-based stochastic optimization techniques such as genetic algorithm (GA) [13] and particle swarm optimization (PSO) [14], [15] have been used increasingly as an effective technique for microarray data analyses [16]–[22]. In [19], [20], binary PSO (BPSO) [23] combined with filter method was applied for searching optimal gene subsets. The method in [19] simplified gene selection and obtained a higher classification accuracy compared to some similar gene selection methods based on GA, while the method in [20] could determine the appropriate number of genes and obtained high classification accuracy by support vector machine. In [21], an approach combined GA with K-nearest neighbor (KNN) method was proposed to identify genes that could jointly discriminate between different classes of samples. The GA/KNN approach could capture the correlated structure in the data and are highly repeatable in independent runs [21]. A combination of Integer-Coded GA (ICGA) and particle swarm optimization, coupled with extreme learning machine (ELM) was used to select an optimal set of genes [22]. These hybrid methods were capable of selecting a compact subset of predictive genes for sample classification. However, these methods considered only the features’ relevance by evaluating their utility for achieving accurate predication or exploiting data variance and distribution, and the selected genes were usually poorly explicable.

Similar to GA, PSO searches for optima by updating population with generations. Unlike GA, PSO has no evolution operators such as crossover and mutation. PSO is easy to implement with few parameters need to adjust. Binary PSO (BPSO) algorithm is a binary version of PSO, which is suitable to solve discrete optimization problems [23].

In the gene selection process, different kinds of classification algorithms such as backpropagation (BP) [24], KNN [25] and support vector machine (SVM) [26], were used to evaluate the prediction ability of gene subsets, which may result into high computational cost in wrapper and embedded methods. The convergence performance, especially convergence rate, of the classification algorithm to evaluate the candidate gene subsets is a significant factor in the gene selection process. ELM, developed recently for a single hidden layered neural network (SLFN) [27], has good generalization performance with a fast training procedure. In ELM, the input weights are chosen randomly and the output weights are calculated analytically. Normally, ELM’s performance is superior to other classifiers such as gradient-based learning algorithms and SVM for problems with larger sample sets and a smaller number of features [22]. Therefore, it is a reasonable choice to use ELM to evaluate the candidate gene subsets.

In Kmeans-PSO-ELM method [28], we used K-means method to group the initial gene pool into several clusters, and standard PSO combined with ELM was used to perform gene selection, which could obtain a compact set of informative genes. Based on the work in [28], a prior information referred to as gene-to-class sensitivity (GCS) is considered in the gene selection process to select smallest possible set of predictive genes with high interpretability in this paper. The GCS information is exploited by SLFN from microarray data. The GCS information, indicating if a gene is sensitive to sample classes, contributes to select those genes significantly correlated to the sample classes. To identify relevant genes effectively for subsequent researches such as sample classification, we partition all genes into different clusters by K-means method, and filter out some clusters and genes with low GCS values. However, some remainder genes, especially those in the same cluster, may have high similarity so that there is probably large with rich redundancy among these remainder genes. To identify small sets of genes that could be used for diagnostic purpose, a modified binary PSO coupling GCS information combined with ELM is used to select smallest possible gene subsets. The modified BPSO may select the representative genes from the remainder clusters to form the optimal gene subset. Moreover, the hybrid method could reduce computational cost for using ELM to evaluate the candidate gene subsets.

## Methods

### K-means Clustering Method

Clustering is a search for hidden patterns that may exist in datasets. It is a process of grouping data objects into disjointed clusters so that the data in each cluster are similar, yet different to the others [29]. There are two kinds of clustering algorithms: hierarchical clustering and partitioned clustering. Different from hierarchical approaches, the partitioned clustering approach divided the input data into specified in advance number of clusters by minimizing a certain cost-function [29].

K-means, a typical partitioned clustering method, is simple and generally very fast [29]. It initializes specified in advance number of centers by some initial values called seed-points. Then, K-means computes the squared distances between the input data points and centers, and assigns the input data points to the nearest centers. Each cluster is represented by an adaptively-changing center. The above process is repeated until all center positions are optimized. The standard K-means algorithm minimizes the square-error cost-function as follows:
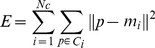
(1)where *p* is a input data point in the cluster *C_i_, i = 1,2,…, N_c_,* and *m_i_* is the center of cluster *C_i_* (the mean of all data points in the cluster *C_i_*).

### Particle Swarm Optimization

PSO is an evolutionary computing mechanism in searching for the best solution by simulating the movement of flocking birds [14]. The population of the birds is called the swarm, and the members of the population are called the particles. Each particle represents a possible solution to the problem. In the process of optimization, each particle flies independently in its own direction which is guided by its individual historical best position (*pbest*) as well as the global best position of all particles (*gbest*). Supposing the dimension of a searching space is *D*, and the swarm is *S = (X_1_,X_2,_X_3_,…,X_ns_)*; each particle stands for a position in *D*-dimensional space; the position of the *i*-th particle in the search space can be denoted as *X_i_ = (x_i1_, x_i2,_…, x_iD_)*, *i* = *1, 2, …, ns*, where *ns* is the swarm size. The individual historical best position of the *i*-th particle is expressed as *P_i_ = (p_i1_,p_i2,_…,p_iD_)*. The global best position of all particles is denoted as *P_g_ =  (p_g1_,p_g2,_…,p_gD_)*. The velocity of the *i*-th particle is expressed as *V_i_ =  (v_i1_,v_i2_,…,v_iD_)*. According to [15], the basic PSO is described as:
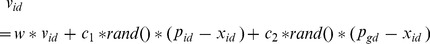
(2)

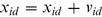
(3)where *c_1_*, *c_2_* are the acceleration constants with positive values; *rand()* is a random number ranged from 0 to 1; *w* is the inertia weight.

The basic PSO algorithm is usually applied to solve problems in which the elements of the solution are continuous real number, whereas BPSO is more suitable for discrete optimization [23]. In BPSO, the velocity update formula remains unchanged as shown in Eq.(2), while the new position of the particle is calculated according to the following equation:
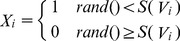
(4)where the function *S(V_i_)* is a sigmoid function, 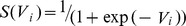.

### Extreme Learning Machine

In [30], a learning algorithm for SLFN called extreme learning machine (ELM) was proposed to solve the problem caused by gradient-based learning algorithms. ELM randomly chooses the input weights and hidden biases, and analytically determines the output weights of SLFN. ELM has much better generalization performance with much faster learning speed than gradient-based algorithms [31], [32].

For *N* arbitrary distinct samples (*XX_i_, T_i_*) (*i = 1,2,…,N.*), where *XX_i_ = *[*xx_i1_, xx_i2_, …,xx_in_*]*∈R^n^*, *T_i_* = [*t_i1_, t_i2_, …,t_im_*]*∈R^m^*. A SLFN with *N_H_* hidden neurons and activation function *g(·)* can approximate these *N* samples with zero error. This means that
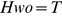
(5)where



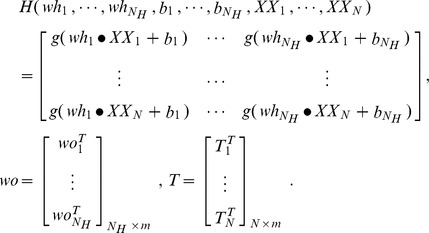



The *wh_i_ = *[*wh_i1_, wh_i2_, …,wh_in_*]^T^ is the input weight vector connecting the *i*-th hidden neuron and the input neurons, the *wo_i_* = [*wo_i1_, wo_i2_, …,wo_im_*] is the output weight vector connecting the *i*-th hidden neuron and the output neurons, and the *b_i_* is the bias of the *i*-th hidden neuron.

In the course of learning, first, the input weights and the hidden biases are arbitrarily chosen and need not be adjusted at all. Second, the smallest norm least-squares solution of the Eq. (5) is obtained as follows:
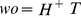
(6)It was concluded that the ELM has the minimum training error and smallest norm of weights [31], [32]. The smallest norm of weights tends to have the best generalization performance [31], [32]. Since the solution is obtained by an analytical method and all the parameters of SLFN need not be adjusted, ELM converges much faster than gradient-based algorithm.

### The Proposed Hybrid Method

#### Gene-to-class sensitivity information

To get a better understanding of gene-to-class sensitivity (GCS) information, the input-to-output sensitivity of a SLFN should be given first. *wh_ki_* is the connected weight from the *i*-th input node to the *k*-th hidden neuron, and *wo_jk_* is the connected weight from the *j*-th hidden neuron to the *k*-th output neuron. *xx_i_* is the *i*-th input component and *O_j_* is the *j*-th output component of the SLFN. The *i*-th input to *j*-th output sensitivity [33], [34] is defined as:
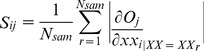
(7)where *N_sam_* is the number of samples. For simplicity, the hidden activation function of the SLFN is *logsig* function, and the output neurons are linear. Obviously, the derivative 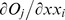 obtained from the *r*-th sample, *XX_r_*, can be calculated as:
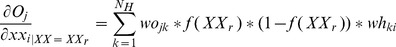
(8)When the *O_j_* is the value of the *j*-th class and *xx_i_* represents the expression level of the *i*-th gene, *S_ij_* in Eq.(7) can be considered as the *i*-th gene to the *j*-th class sensitivity. The larger the *S_ij_* value is, the more sensitive to the *j*-th class the *i*-th gene is. The GCS value for the *k*-th gene is normalized as follows:
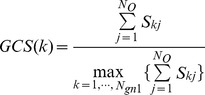
(9)where *N_O_* and *N_gn1_* are the number of the output neurons in the SLFN and the number of genes in the first-level initial gene pool.

In this study, a SLFN is trained with the training dataset by ELM, and the input and output weights of the SLFN are determined. According to Eqs. (7)–(9), the GCS values of all genes are easily obtained. To obtain more accurate GCS value of each gene, the above operation is repeated 50 times and the average GCS value of each gene is calculated.

### The Proposed Gene Selection Method

**Figure 1:**
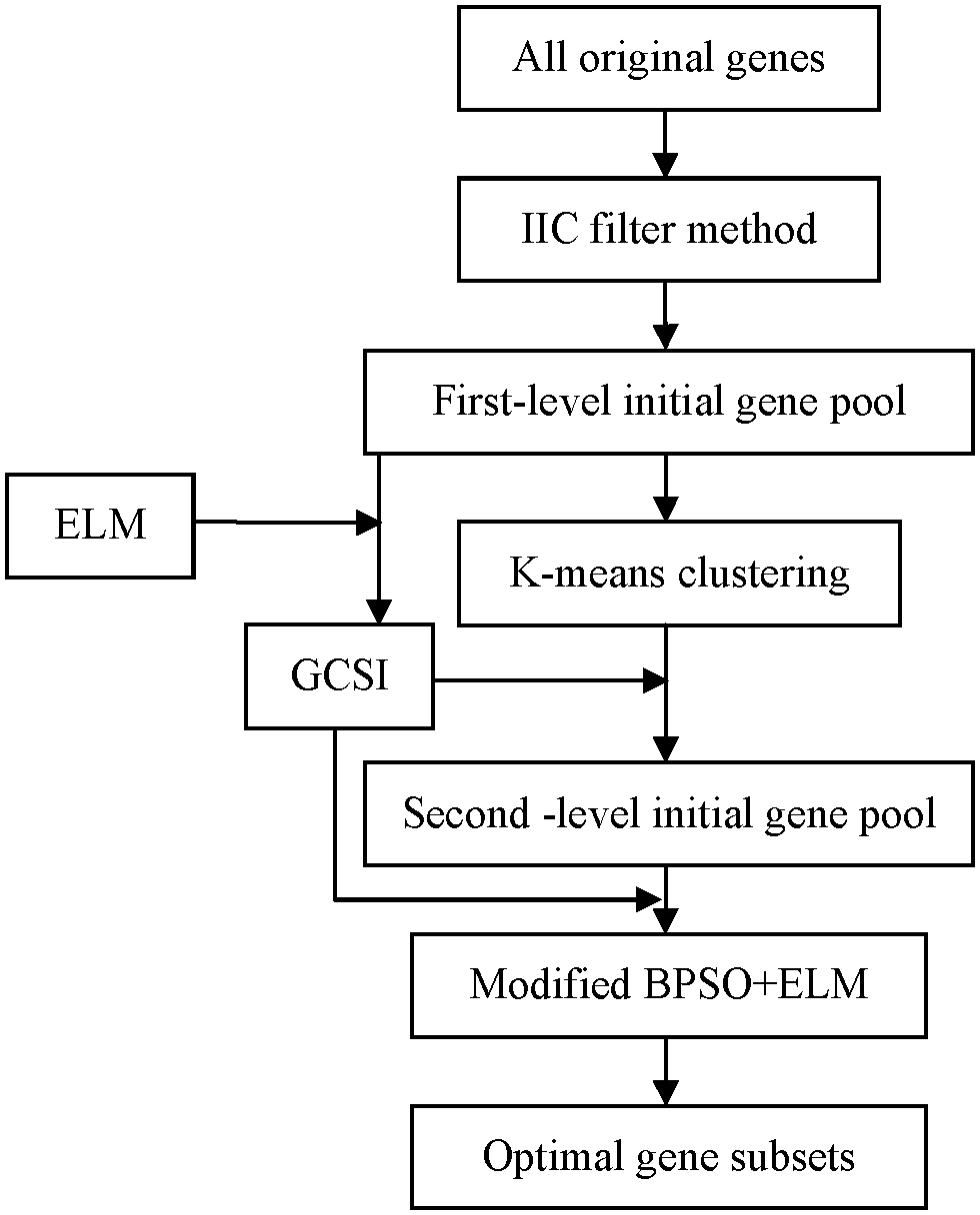
The frame of the proposed hybrid gene selection method.

In this paper, gene selection also consists of two respects, which are to identify relevant genes from each cluster and to tend to select smallest subsets from the relevant genes. To obtain compact and explicable gene subsets, GCS information (GCSI) exploited from microarray data is considered in the whole process of gene selection. The rough frame of the proposed method is shown in Figure 1.

To identify relevant genes for subsequent sample classification, firstly, the GCS values of all genes are calculated according to Eqs.(7)–(9), and all genes are clustered by K-means algorithm. Since the genes in the same cluster might have similar functions, it is possible that several genes serve as the equivalent for the subsequent sample classification. To reduce the redundancy, only the representative genes among these equivalent genes are selected for further processing. Then, if maximum GCS value of all genes in a cluster is far lower than those of other clusters, this cluster will be filtered out. Moreover, if the GCS value of a gene in the remainder cluster is lower than the mean of the GCS values of all genes in this cluster, this gene will also be filtered out. Thus, the remainder genes in the remainder clusters have comparatively high GCS values, and these relevant genes for sample classification are probably kept for further gene selection.

Although the remainder genes are relevant to data classes, there is still rich redundancy among these genes. In this study, a modified BPSO encoding GCS information is proposed to select the most compact gene subsets from the remainder genes. The detailed steps are described as follows.

Step 1: Form a first-level initial gene pool. The dataset is divided into training and testing datasets. Select 200∼400 genes from all original genes by using information index to classification (IIC) method [35] on the training data. Since the original IIC method is used for two-class microarray data, we develop it for multi-class microarray data as follows:
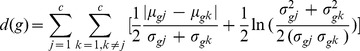
(10)where 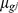 and 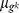 are the means of expression value of the gene *g* in the *j*-th and *k*-th classes, respectively, and 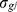 and 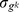 are the standard deviations of expression value of gene *g* in the *j*-th and *k*-th classes, respectively. *c* is the total number of classes. From [35], the higher the value of *d(g)*, the more classification information the gene *g* contains, so the gene *g* is more relevant to samples categories. The high classification accuracy will be obtained with high probability by a classifier if the microarray data is projected onto the gene *g* whose IIC value, *d(g)*, is high. The genes are ranked by their IIC values on the training dataset, and those genes with higher IIC values are chosen to establish the first-level gene pool.

Step 2: Establish a second-level initial gene pool based on the first-level initial gene pool. Cluster the genes in the first-level initial gene pool to predetermined number of groups with K-means method. The predetermined number of the clusters is determined by trial and error. Delete those clusters whose maximum GCS values are much smaller than other clusters. Moreover, in each remainder cluster, the genes whose GCS values are smaller than the mean GCS value of all genes in the cluster are also removed. All the remainder genes in the remainder clusters form a second-level initial gene pool to perform further gene selection.

Step 3: Use BPSO to select the optimal gene subsets from the second-level initial gene pool. To improve the search ability of the swarm, GCS information is encoded into BPSO for further gene selection.

Firstly, initialize a population of particles with random positions and velocities in the search space. The *i*-th particle *X_i_ = (x_i1_, x_i2_, …,x_iD_)* represents a candidate gene subset, and the element *x_ij∈{0,1},(1≤j≤D)_* indicates whether the *j*-th gene is selected. The dimension of a particle is equal to the number of genes in the second-level initial gene pool. Since the number of the remainder clusters is comparatively small and each cluster owns its representativeness, at least one gene should be selected from each cluster in the initialization of each particle.

Secondly, update the position of each particle. To encode GCS information, two modified equations for updating the particles based on the discrete PSO in [20] are proposed as follows:
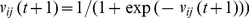
(11)

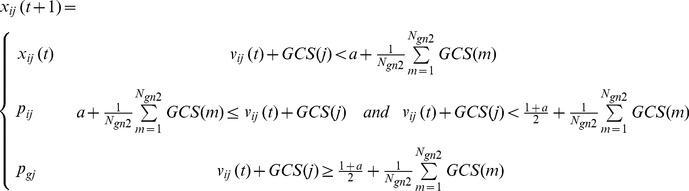
(12) where *GCS(j)* is the GCS value of the *j*-th gene, and *N_gn2_* is the number of genes in the second-level initial gene pool. *v_ij_(t+1)* on the right-hand side of Eq.(11) is calculated by Eq.(2). The parameter, *a*, is a fixed number, and is set as 0.5 according to [20]. The subscripts *i* and *j* denote the *i*-th particle and its *j*-th component. The *p_ij_* is the *j*-th component of the historical best position of the *i*-th particle, and the *p_gj_* is the *j*-th component of the global best position of all particles. The above equations make the genes with high GCS values be selected with high probability.

Thirdly, evaluate the fitness value of each particle, and update the historical best position of each particle and the global best position of all particles. To select smallest possible predictive gene subsets, the fitness function is defined as follows:
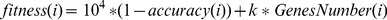
(13)According to [36], to correct for the selection bias in the gene selection process, we perform 5-fold cross validation (CV) to evaluate the selected genes. The *accuracy(i)* in Eq. (13) is the 5-fold cross validation accuracy on training data obtained by ELM with the candidate genes denoted by the *i*-th particle. The *GenesNumber(i)* is the number of the selected genes denoted by the *i*-th particle.

Finally, if the fitness value reaches the threshold value, or the maximal iterative generations are arrived, the particle with the best fitness value is output. So the optimal gene subset is obtained.

As a wrapper or embedded gene selection method, its main computational cost is the computational time of evaluating the candidate gene subset by classifier. In the proposed method, the classifier to evaluate the candidate gene subsets is ELM which is trained by an analytical method without iterations. In those gene selection methods using SVM or BP algorithm to evaluate the candidate gene subsets, the classifier is trained with thousands of iterations and the training process is very time consuming. Therefore, the computational time of the proposed method is much less than that of methods using SVM or BP to evaluate the candidate gene subset. Moreover, for better generalization performance of ELM, the final selected gene subset evaluated by ELM has high predictive ability. The proposed method combines K-means method with the modified BPSO, GCS information and ELM, so it is referred to as KMeans-GCSI-MBPSO-ELM.

## Results and Discussion

### Datasets

To verify the effectiveness and efficiency of the proposed gene selection method, we conduct experiments on six open microarray datasets including Leukemia, Colon, SRBCT, LUNG, Brain cancer and Lymphoma data. The detailed description of the datasets is listed in Table 1.

**Table 1:** Six microarray data.

Data	Total	Training	Testing	Classes	Genes
Leukemia	72	38	34	2	7129
Colon	62	40	22	2	2000
SRBCT	83	63	20	4	2308
LUNG	203	103	100	5	3312
Brain cancer	60	30	30	2	7129
Lymphoma	58	29	29	2	7129

The Leukemia data [37] contains total 72 samples in two classes, acute lymphoblastic leukemia (ALL) and acute myeloid leukemia (AML), which contain 47 and 25 samples, respectively. Every sample contains 7129 gene expression values. The Leukemia data are available at http://www-genome.wi.mit.edu/cgi-bin/cancer/datasets.cgi.

The Colon data consists of expression levels of 62 samples of which 40 samples are colon cancer samples and the remaining are normal samples. Although original expression levels for 6,000 genes are measured, 4,000 genes out of all the 6,000 genes were removed considering the reliability of measured values in the measured expression levels. The measured expression values of 2,000 genes are publicly available at http://microarray.princeton.edu/oncology/.

The entire SRBCT data [33] includes the expression data of 2308 genes. There are totally 63 training samples and 25 testing samples, five of the testing samples being not SRBCT. The 63 training samples contain 23 Ewing family of tumors (EWS), 20 rhabdomyosarcoma (RMS), 12 neuroblastoma (NB), and 8 Burkitt lymphomas (BL). The 20 testing samples contain 6 EWS, 5 RMS, 6 NB, and 3 BL. The data are available at http://www.biomedcentral.com/content/supplementary/1471-2105-7-228-S4.tgz.

The LUNG data [38], [39] contains in total 203 samples in five classes, adenocarcinomas, squamous cell lung carcinomas, pulmonary carcinoids, small-cell lung carcinomas and normal lung, which have 139, 21, 20, 6,17 samples, respectively. Each sample has 12600 genes. The genes with standard deviations smaller than 50 expression units were removed and a dataset with 203 samples and 3312 genes was obtained [38], [39]. The data is also available at http://www.biomedcentral.com/content/supplementary/1471-2105-7-228-S4.tgz.

The Brain cancer data contains 60 samples in two classes, 46 patients with classic and 14 patients with desmoplastic brain cancer. The Lymphoma data includes 58 samples where 32 patients did cured and 26 patients did not cured. Each sample in the Brain cancer and Lymphoma has 7129 genes. These two data are available at http://linus.nci.nih.gov/~brb/DataArchive_New.html.

Since there has no guidance on how to select the population size and maximum iteration number in PSO, we determine the values of these parameters within the crossvalidation runs on the validation dataset. As for using modified BPSO to select optimal genes, the population size is 100 on the SRBCT data, while the one is 50 on the other five data; the maximum iteration number is 20 on the SRBCT, LUNG and Brain cancer data, and the one is 40 on the Leukemia, Colon and Lymphoma data. According to [15], a recommended choice for the acceleration constants *c_1_* and *c_2_* is 2, and a better decrease for the inertia weight, *w*, is form 1.4 to 0.5. In this study, based on the conclusions in [15] and the crossvalidation runs on the validation dataset, the initial and final inertia weight are set as 1.2 and 0.4, respectively on all data, and the acceleration constants *c_1_* and *c_2_* are both selected as 1.6 on all data.

### Reduce Redundant Genes

**Figure 2:**
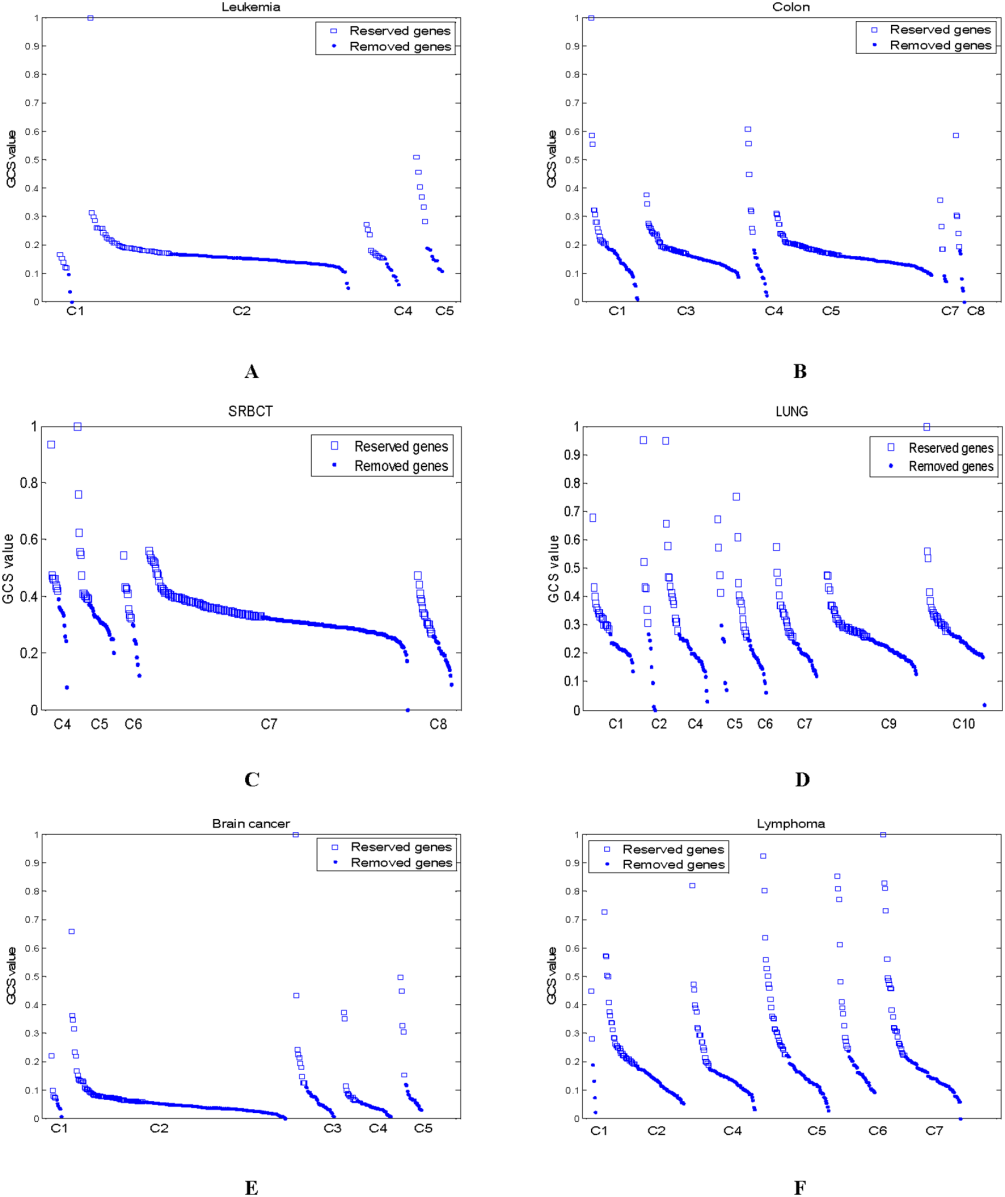
The GCS values of genes in the remainder clusters (The character ‘C’ along the X axis is the abbreviation for ‘Cluster’) (A) Leukemia (B) Colon (C) SRBCT (D) LUNG (E) Brain cancer (F) Lymphoma.

In the first-level initial gene pool, some clusters with low maximum GCS values are deleted. In the experiments, Cluster 3 is removed on the Leukemia data, Clusters 2 and 6 are deleted on the Colon data, Clusters 1, 2 and 3 are deleted on the SRBCT data, Clusters 3 and 8 are filtered out on the LUNG data, Cluster 6 are deleted on the Brain cancer data, and Cluster 3 are filtered out on the Lymphoma data. Moreover, some genes in a remainder cluster whose GCS values are lower than the mean GCS values of the cluster are removed. Figure 2 shows the GCS values of the genes in the remainder clusters on all data. From Figure 2, over half the genes in the remainder clusters are filtered out. The less the reserved genes are, the less the computational cost of the further gene selection is.

### The Classification Ability of the Selected Gene Subsets

To verify the classification ability of the selected gene subsets, ELM is used to perform sample classification with these selected gene subsets on the six datasets. Each experiment is conducted 100 times, and the mean results are listed in Table 2.

**Table 2:** The classification accuracies with different gene subsets by ELM on the six data.

Data	Selected genes	5-fold CV Accuracy Mean(%)±std	Testing Accuracy Mean(%)±std
Leukemia	2642,4050, 2121	100±0.00	100±0.00
	2642,4050,3258	100±0.00	100±0.00
	2642,4050,1882	99.74±0.55	100±0.00
	4050,1685,1078,2121	99.64±0.61	98.21±1.61
Colon	141,792,251,1679,1976,14	97.61±1.37	93.68±2.58
	141,1110,792,251,1976,286, 23,14	98.03±1.46	94.36±2.59
	127,652,1110,43,251,1976, 795,1071,286,14	97.50±1.63	95.09±3.27
	304,360,377,1110,792,312,251,36,1763,1867,1976,795,14	98.05±1.38	96.14±2.53
SRBCT	742,1003,1055,2050,846, 1772	100±0.00	100±0.00
	742,1003,603,971,846,1389	100±0.00	100±0.00
	236,976,1003,123,819,545	100±0.00	100±0.00
	742,1003,2050,235,1634,1120, 545	100±0.00	100±0.00
LUNG	498,614,567,2750,1209, 1765,2763,867,2659,2670	96.88±0.61	94.80±0.79
	641,777,1288,614,567,320, 3178,792,3295,2558,997	97.10±0.63	93.02±1.17
	580,103,2750,1559,1765,2763,2583,997,1014	96.17±0.65	94.44±0.82
Brain cancer	3362,1970,3123,5931	86.07±1.99	77.20±1.40
	6571,4413,4917,5931	85.70±3.16	79.53±3.96
	5721,4069,1970,3123,5931	87.87±1.73	78.30±1.74
	6571,4409,4413,4628,1970,5931	88.63±2.16	80.40±3.36
Lymphoma	4092,6171,412,5843,806,4037	85.05±2.44	78.62±1.70
	5660,4092,364,152,956,806,4037	84.60±2.75	74.52±2.40
	4092,6171,5357,3646,5909,152,806,2650	86.97±2.44	73.38±3.10
	5660,4092,6171,510,6219,2374,1568,2650	86.95±2.33	78.79±1.24

From Table 2, with the three genes selected by the proposed approach, ELM obtains 100% testing accuracy and 5-fold CV testing accuracy on Leukemia. ELM obtains 100% testing accuracy and 5-fold CV testing accuracy with all selected gene subsets on the SRBCT data. With the about ten genes selected by the KMeans-GCSI-MBPSO-ELM method, ELM obtains high prediction accuracies on the other four data. These results indicate that the KMeans-GCSI-MBPSO-ELM method has the ability of selecting most predictive genes highly related to sample classes.

**Figure 3:**
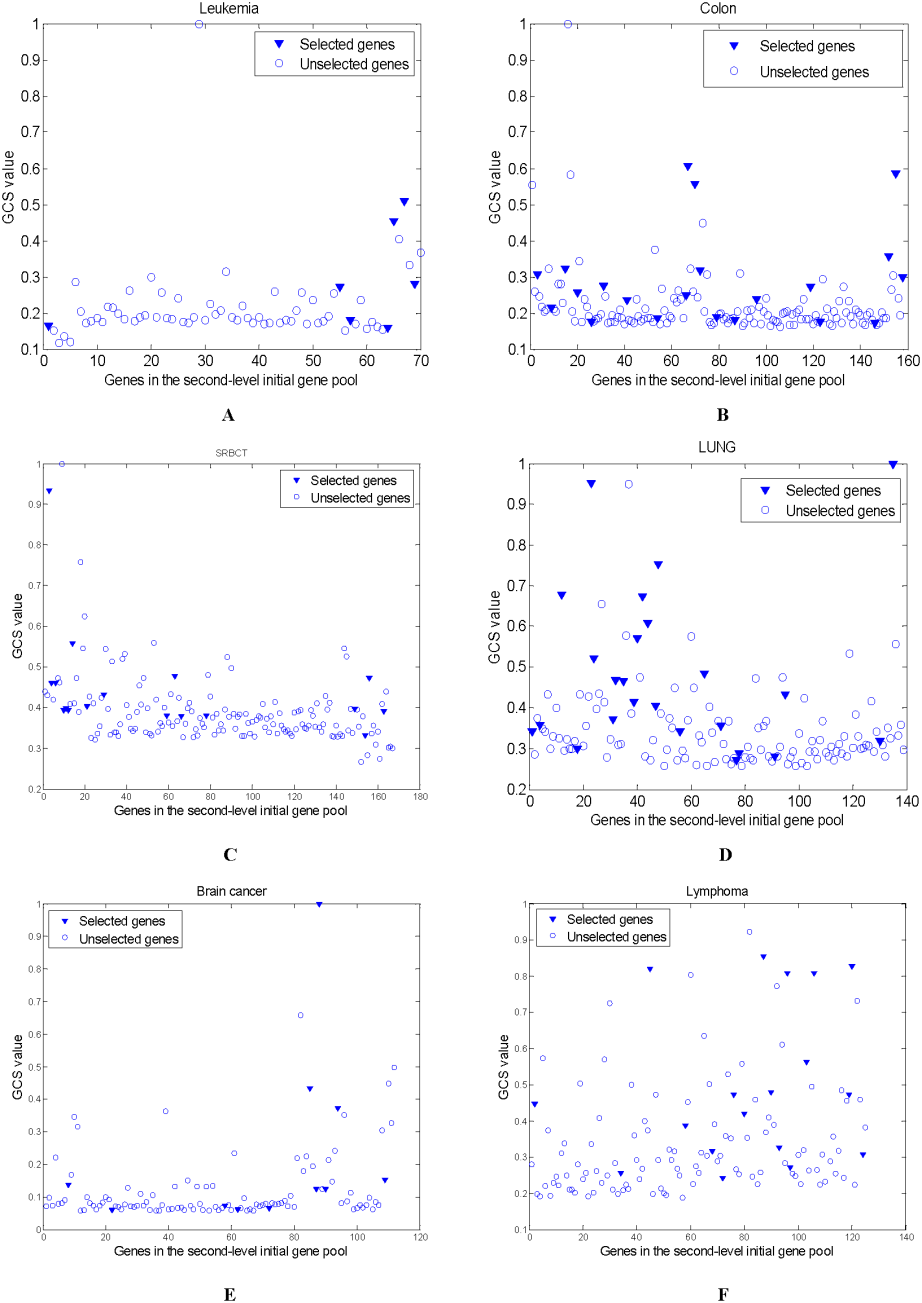
The GCS values for all reserved genes (A) Leukemia (B) Colon (C) SRBCT (D) LUNG (E) Brain cancer (F) Lymphoma.

The GCS values of all reserved genes listed in Figure 2 are shown in Figure 3. It can be found that the KMeans-GCSI-MBPSO-ELM method does not always select those genes with the highest GCS values, and it also selects those critical genes with comparatively low GCS values. This is mainly because the modified BPSO considers not only the GCS values of the selected genes but also the classification ability of each gene subset.

### Biological and Functional Analysis of the Selected Gene Subsets

The experiment on each microarray data is conducted 1000 times, and the top ten frequently selected genes are listed in Tables 3–8 for the six data.

**Table 3:** The top ten frequently selected genes with the proposed method on the Leukemia data.

Gene no.	Gene name	Description
2642	U05259	MB-1 gene*#
4050	X03934	GB DEF = T-cell antigen receptor gene T3-delta
2121	M63138	CTSD Cathepsin D (lysosomal aspartyl protease)*#
3320	U50136	Leukotriene C4 synthase (LTC4S) gene*#
6539	X85116	Epb72 gene exon 1*#
1882	M27891	CST3 Cystatin C (amyloid angiopathy and cerebral hemorrhage)*#
5191	Z69881	Adenosine triphosphatase, calcium#
1779	M19507	MPO Myeloperoxidase*#
4847	X95735	Zyxin*#
1078	J03473	ADPRT ADP-ribosyltransferase (NAD+; poly (ADP-ribose) polymerase)

*Also selected by the method in [40].

#Also selected by the method in [37].

**Table 4:** The top ten frequently selected genes with the proposed method on the Colon data.

Gene no.	Gene name	Description
14	H20709	Myosin light chain alkali, smooth-muscle isoform (Human) ♀♂
237	T50334	14-3-3-like protein GF14 omega (Arabidopsis thaliana)
1482	T64012	Acetylcholine receptor protein, delta chain precursor (Xenopus laevis)
175	T94579	Human chitotriosidase precursor mRNA, complete cds. ♂
286	H64489	Leukocyte antigen CD37 (Homo sapiens)
141	D21261	Sm22-alpha homolog (Human)
792	R88740	Atp synthase coupling factor 6, mitochondrial precursor (Human) ♂
3	R39465	Eukaryotic initiation factor 4A (Oryctolagus cuniculus)
251	U37012	Human cleavage and polyadenylation specificity factor mRNA, complete cds
23	R22197	60S ribosomal protein L32 (Human) ♀

♀ Also selected by the method in [41].

♂ Also selected by the method in [42].

**Table 5:** The top ten frequently selected genes with the proposed method on the SRBCT data.

Gene no.	Gene name	Description
1003	796258	sarcoglycan, alpha (50kD dystrophin-associated glycoprotein)† ‡
742	812105	transmembrane protein†
1601	629896	microtubule-associated protein 1B† ‡
603	42558	glycine amidinotransferase (L-arginine:glycine amidinotransferase)†
1055	1409509	troponin T1, skeletal, slow† ‡
545	1435862	antigen identified by monoclonal antibodies 12E7, F21 and O13† ‡
1955	784224	fibroblast growth factor receptor 4† ‡
1	21652	catenin (cadherin-associated protein), alpha 1 (102 kD)† ‡
1389	770394	Fc fragment of IgG, receptor, transporter, alpha† ‡
976	786084	chromobox homolog 1 (Drosophila HP1 beta)

† Also selected by the method in [33].

‡ Also selected by the method in [43].

**Table 6:** The top ten frequently selected genes with the proposed method on the LUNG data.

Gene no.	Gene name	Description
498	39755	Cluster Incl Z93930:Human DNA sequence from clone 292E10 on chromosome 22q11–12. Contains
1559	1011_s	tyrosine 3-monooxygenase/tryptophan 5-monooxygenase activation protein, epsilon polypeptide
792	38704	actin binding protein; macrophin (microfilament and actin filament cross-linker protein)
3178	38799	Cluster Incl AF068706:Homo sapiens gamma2-adaptin (G2AD) mRNA, complete cds/cds = (763,3018)
1765	39722	nuclear receptor co-repressor 1
1243	39012_g	endosulfine alpha
614	1147	V-Erba Related Ear-3 Protein
2750	38484	synaptosomal-associated protein, 25 kD
1014	588	protein tyrosine phosphatase, non-receptor type 1
567	33412	Cluster Incl AI535946:vicpro2.D07.r Homo sapiens cDNA, 5 end/clone_end = 5″/gb = AI535946

**Table 7:** The top ten frequently selected genes with the proposed method on the Brain cancer data.

Gene no.	Gene name	Description
5931	X58987	dopamine receptor D1
4413	U39817	Bloom syndrome
130	AFFX-BioDn-5_st	/
1745	L08895	MADS box transcription enhancer factor 2, polypeptide C (myocyte enhancer factor 2C)
6732	Y00317	UDP glucuronosyltransferase 2 family, polypeptide B4
4843	U61262	neogenin homolog 1 (chicken)
2935	M60459	erythropoietin receptor
3502	S74683	ADP-ribosyltransferase 1
1970	L25270	Smcy homolog, X-linked (mouse)
18	AB000895	dachsous 1 (Drosophila)

**Table 8:** The top ten frequently selected genes with the proposed method on the Lymphoma data.

Gene no.	Gene name	Description
5660	X14046	CD37 antigen
1116	HG2815-HT4023_s	/
2748	M33478	phosducin
5357	U90543	butyrophilin, subfamily 2, member A1
806	D86969	PHD finger protein 16
3823	U09770	cysteine-rich protein 1 (intestinal)
4269	U32324	interleukin 11 receptor, alpha
5381	U90914	carboxypeptidase D
2073	L36033	chemokine (C-X-C motif) ligand 12 (stromal cell-derived factor 1)
6105	X67098	enolase superfamily member 1

From Table 3, all genes except genes X03934, Z69881 and J03473 were also selected by the methods proposed in [40] and [37]. Gene Z69881 is also selected by the proposed method in [37]. Gene U05259, a B lymphocyte antigen receptor, encodes cell surface proteins for which monoclonal antibodies have been demonstrated to be useful in distinguishing lymphoid from myeloid lineage cells [40]. Gene M63138 is the member of the peptidase C1 family involved in the pathogenesis of breast cancer and possibly Alzheimer’s disease [40]. Gene X95735, an AML-related gene, is an adhesive plaque protein, which plays a central role in regulation of cell differentiation [40]. Form Tables 2 and 3, it can be concluded that gene X03934 is also a critical gene for sample classification.

From Table 4, genes H20709 and R22197 were also selected in [41], and genes H20709, T94579 and R88740 were also selected in [42]. A muscle index can be calculated based on an average intensity of 17 ESTs in the array that are homologous to smooth muscle genes which included gene H20709 [41]. Gene R22197 is one of the ribosomal protein cluster which are ESTs homologous to genes that appear to be related to cellular metabolism such as an ATP-synthase component and an elongation factor [41].

From Table 5, genes 796258, 629896, 1409509, 1435862, 784224, 21652 and 770394 were also both selected by the methods proposed in [33] and [43]. Genes 812105 and 42558 were also selected by the method proposed in [33]. Some genes are over-expressed in a certain type of tumor but lack of specificity. For instance, Gene 784224 (fibroblast growth factor receptor 4) was noted to be highly expressed only in RMS and not in normal muscle, but it is also expressed in some other cancers and normal tissues [33]. This tyrosine kinase receptor is expressed during myogenesis but not in adult muscle, and is of interest because of its potential role in tumor growth and in prevention of terminal differentiation in muscle [33].

Although many gene selection methods were used on the LUNG, Brain cancer and Lymphoma data, they did not give the detailed information of the selected genes [38], [39], [44], [45], and thus we do not know which genes listed in Tables 6–8 were also selected by these methods. Genes listed in Tables 6–8 can be a useful reference for future study of these three data.

**Figure 4:**
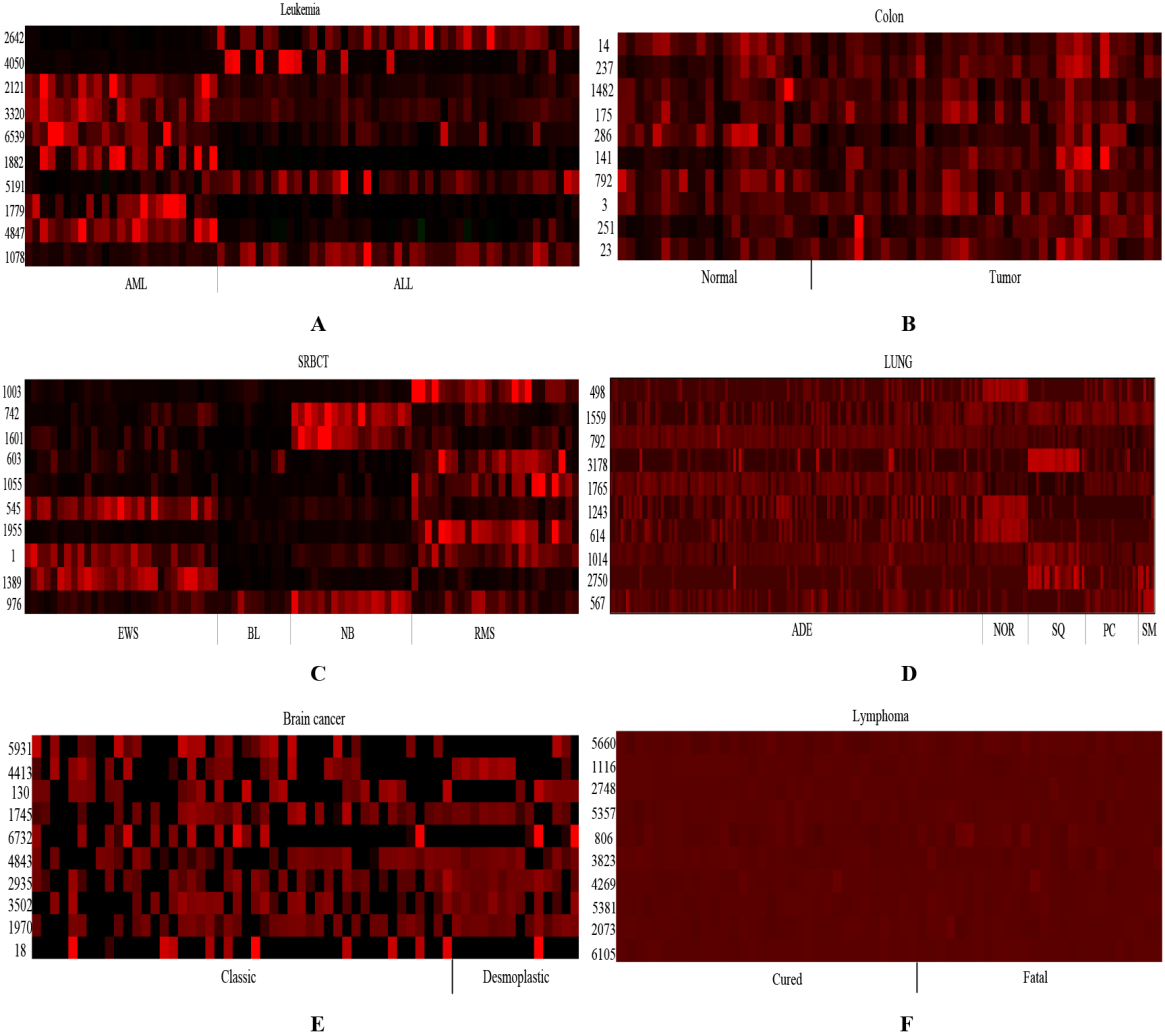
The Heatmap of expression levels based on the top ten frequently selected genes on the six data (A) Leukemia (B) Colon (C) SRBCT (D) LUNG (E) Brain cancer (F) Lymphoma.

To further verify that the proposed method is capable of selecting predictive genes, the heatmap with top ten frequently selected genes for the six data are shown in Figure 4. From Figure 4 (A), all ten genes expression levels clearly differentiate between AML and ALL. From Figure 4 (B), the expression levels of genes 237, 286 and 141 are distinct in two classes. From Figure 4 (C), some genes are over-expressed in a certain type of tumor but lack specificity. For instance, the expression levels of genes 1601 and 742 are distinct from NB and other classes, while the ones of genes 1003, 603, 1055 and 1955 are distinct from RMS and other classes. Gene 545 has different expression level in EWS, and expression level of gene 1 clearly differentiate in BL and NB from EWS and RMS. From Figure 4 (D), the expression levels of genes 498, 1243 and 614 clearly differentiate between NOR and other four classes, while the ones of gene 3178, 1765, 1014, 2750 and 567 differentiate between SQ and other classes. From Figure 4 (E–F), there has no single gene in the Brain cancer and Lymphoma data (especially in the Lymphoma data) whose expression levels are distinct between two classes. This is mainly because all genes in these two datasets have low deviation over all samples.

**Figure 5:**
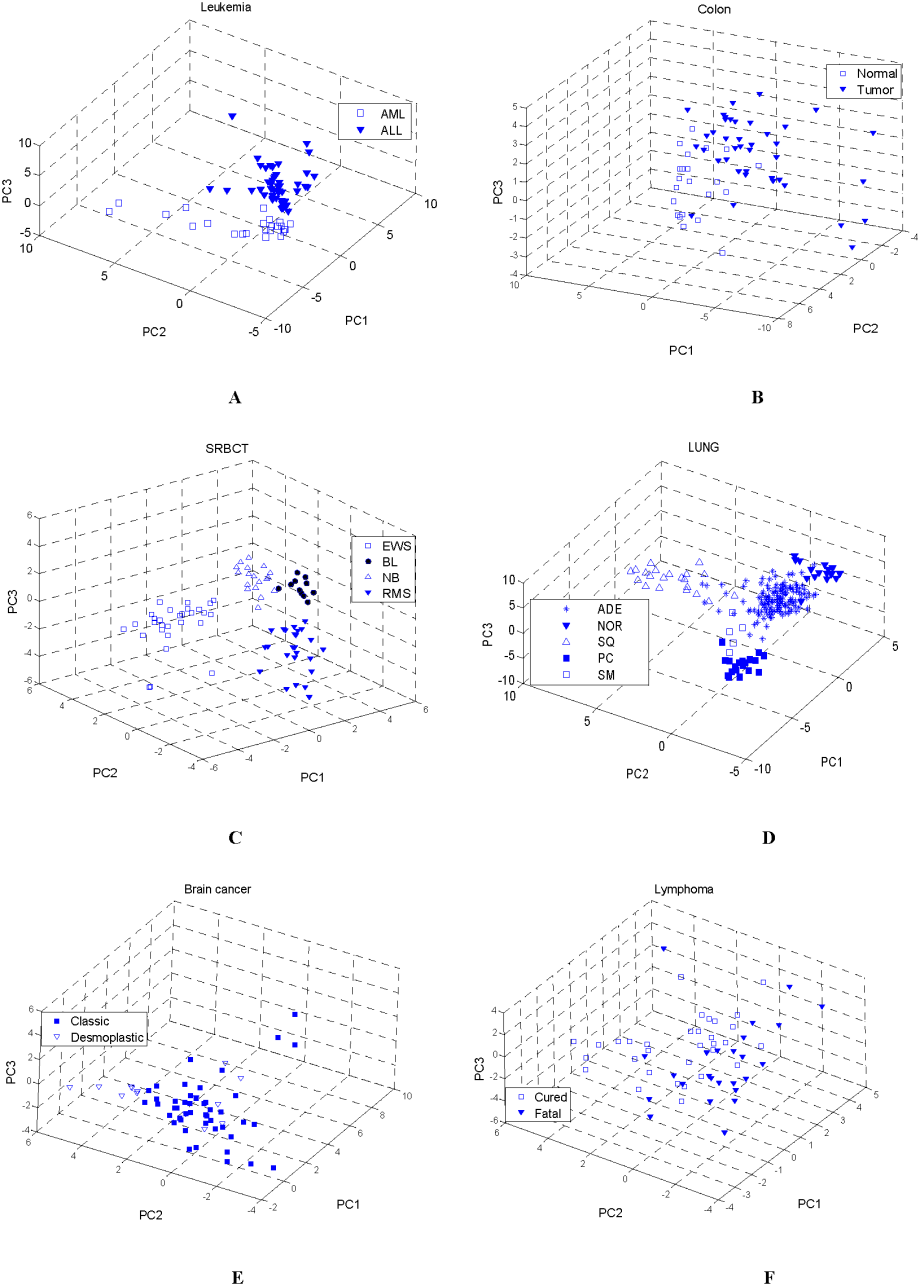
Plot of the first three principal components using the top 30 frequently selected genes (A) Leukemia (B) Colon (C) SRBCT (D) LUNG (E) Brain cancer (F) Lymphoma.

Principal component (PC) analysis is a linear orthogonal transform such that the coordinates of the data in the new space are uncorrelated and the most amount of variance of the original data is preserved by only a few coordinates [46]. Hence, the first few PCs explain most of the variance in the data. A plot of the first three PCs often reveals patterns in the data [47]. Here it is used to project samples of high dimensions onto a three-dimensional plot for visual display. PC analysis is applied to all samples in the six data using the top 30 frequently selected genes, and the display is shown in Figure 5. Noticeably, two and four distinct clusters emerge for the Leukemia and SRBCT data, respectively (Figure 5(A) and (C)). As for the LUNG data (Figure 5(D)), although several samples of different classes are overlapped, five clusters are apparent. As for the Colon data (Figure 5(B)), four normal samples are in the cluster of the tumor samples, which were considered as the contaminated samples in [47]. From Figure 5 (E–F), a few samples of different classes are overlapped. This further indicates that the samples from different classes are not very distinct in both the Brain cancer and Lymphoma data, which was verified in [44], [45].

### Reproducibility of the Proposed Method

**Figure 6:**
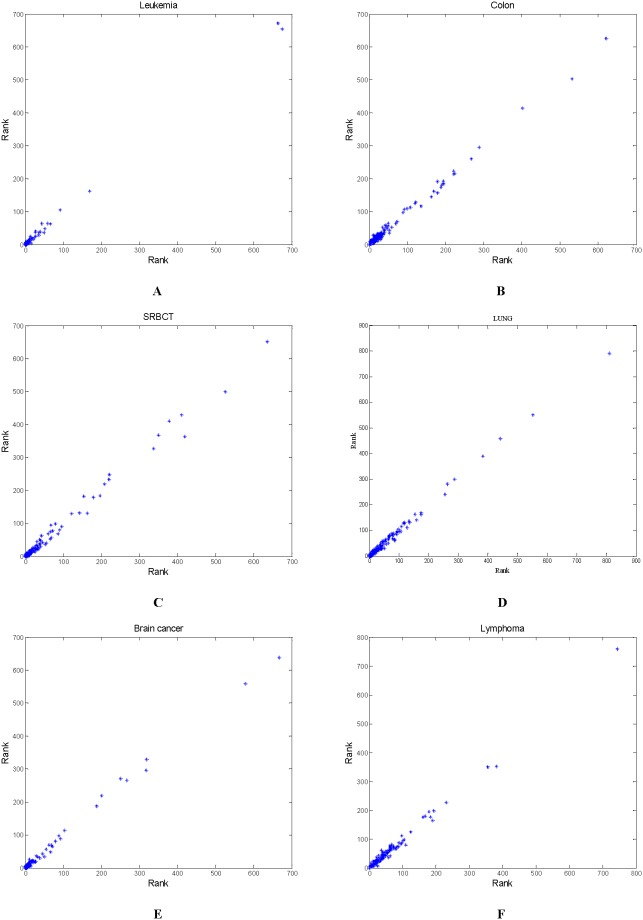
The correlation between the ranks of genes from two independent runs on the six data to assess reproducibility of the proposed approach (A) Leukemia (B) Colon (C) SRBCT (D) LUNG (E) Brain cancer (F) Lymphoma.

To examine the reproducibility of gene selection [21], we repeat the same proposed approach on the six data with different random seed numbers. Another 1000 subsets of optimal particles are thus obtained through an independent run. This means that the proposed method runs 1000 times in one independent run to obtain 1000 optimal gene subsets. All genes in the second-level initial gene pool are ranked according to the frequency of being selected in 1000 solutions obtained by the independent run. Figure 6 depicts the correlation between the ranks from the two independent runs. The points in Figure 6 are distributed along the line, *y = x*, which indicates the proposed method is highly repeatable in two independent runs, so the reproducibility of KMeans-GCSI-MBPSO-ELM is high.

### Convergence Rate of the Proposed Method

**Figure 7:**
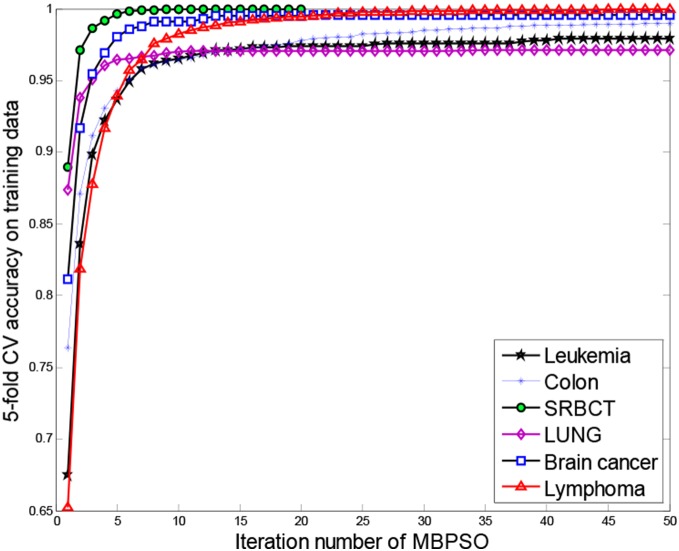
5-fold CV accuracy on the training data versus the iteration number of the MBPSO.

Figure 7 shows the 5-fold CV accuracy on the training data versus the iteration number of the modified BPSO. From Figure 7, the MBPSO finds the optimal gene subset with only five epochs on the SRBCT and LUNG data, 13, 23, 13 and 25 epochs on the Leukemia, Colon, Brain cancer and Lymphoma data, respectively. For the low redundancy of the second-level initial gene pool and encoding the GCS information into BPSO, the modified BPSO could find the optimal gene subset with fast convergence rate. Figure 7 could give a guide for selecting the maximum iteration number in the modified BPSO for different types of microarray data.

### Comparison with Other Gene Selection Methods

First, to compare the KMeans-GCSI-MBPSO-ELM method to other PSO-based gene selection methods such as BPSO-ELM, KMeans-BPSO-ELM and our previous work in [28], ELM is used to perform sample classification with the selected genes obtained by the four gene selection methods. In the BPSO-ELM method, BPSO search the optimal gene subsets and ELM is used to evaluate the gene subsets. As for the KMeans-BPSO-ELM method, all genes are grouped by K-means clustering method first and BPSO combined with ELM to perform gene selection. Compared to the KMeans-GCSI-MBPSO-ELM method, KMeans-BPSO-ELM method does not consider GCS information and not reduce redundant genes in each cluster before using BPSO-ELM to select genes. Each experiment is conducted for 100 times, and the corresponding results of 5-fold CV are listed in Table 9. With the genes selected by the KMeans-GCSI-MBPSO-ELM method, ELM obtains the highest 5-fold CV classification accuracy on all data, whereas it obtains the lowest 5-fold CV classification accuracy on all data with the genes selected by the BPSO-ELM. Although all the four methods combine PSO with ELM to select informative gene subsets, the KMeans-GCSI-MBPSO-ELM method has the best performance because it uses the GCS information to reduce the redundancy of genes and select those gene subsets highly correlated to sample classes.

**Table 9:** The 5-fold CV classification accuracies of the ELM classifier based on four PSO-based gene selection methods on the six data.

Method	Mean accuracy (%)+std. (Gene number)					
	Leukemia	Colon	SRBCT	LUNG	Brain cancer	Lymphoma
KMeans-GCSI-MBPSO-ELM	100±0.00 (3)	97.61±1.37(6)	100±0.00 (6)	97.10±0.63 (11)	88.63±2.16(6)	86.97±2.44(8)
KMeans-BPSO-ELM	99.17±1.04(4)	93.50±2.02(9)	99.27±0.82(7)	95.64±0.56(12)	87.23±2.34(8)	85.14±2.87(6)
BPSO-ELM	98.56±0.27(5)	93.34±1.99(9)	99.82±0.60(10)	94.80±0.57(11)	85.45±2.33(7)	83.50±2.72(8)
Method in [28]	99.64±0.67(3)	93.94±1.17(5)	99.39±0.88(6)	95.67±0.72(11)	86.55±2.35(5)	83.72±2.33(6)

Then, to compare the proposed method with GS1 [38], GS2 [38], Cho’s [48], F-test [49] and the method in [28], we have adopted two ways to build a classifier more than ELM using the top frequently selected genes, one is through support vector machines (SVMs) [50] and the other is through K-nearest-neighbor (KNN) search [49]. As in [38], we also chose a linear kernel in SVM and chose the Euclidean distance in KNN classifier with *K = 5*. To be consistent with [38], each experiment is conducted 100 times on the Leukemia, SRBCT, LUNG and Colon data, and the mean 5-fold CV classification accuracies for three numbers of top ranked genes (that is, 30, 60, and 100) on the Leukemia, SRBCT, LUNG and Colon data are listed in Tables 10–13. The results of GS1, GS2, Cho’s and F-test are directly quoted from [38], and thus the results of these four methods on the Colon data are not provided in Table 13. Since the number of the reserved genes for further gene selection by BPSO in the proposed method is less than 100 on the Leukemia data, the results with 100 genes for six methods are not provided in Table 10. Similarly, the results with 60 genes on the Leukemia data for the method in [28] are not provided. In all cases except the KNN classifier with top 60 frequently selected genes on the Leukemia data, the KNN and SVM classifiers achieve higher classification accuracies with the genes selected by the KMeans-GCSI-MBPSO-ELM method than with the genes selected by other methods but the method in [28]. Moreover, the standard deviations in all cases except the method in [28] on the LUNG data of the KMeans-GCSI-MBPSO-ELM method are always less than those of other gene selection methods, which shows that the proposed method is most robust in most cases. Compared to the proposed method, the KNN and SVM classifiers achieve almost high classification accuracies on the SRBCT and LUNG data with the 60 and 100 genes selected by the method in [28], which indicates the method in [28] requires a comparatively large set including those predictive genes selected by the proposed method.

**Table 10:** The 5-fold CV classification accuracies of the KNN-classifier and SVM-classifier based on six gene selection methods on the Leukemia data.

Method	KNN			SVM		
	30	60	100	30	60	100
New method	96.53±1.16	95.97±1.40	/	98.42±0.48	98.40±0.54	/
Method in [28]	94.04±1.79	/	/	95.83±1.84	/	/
GS2	96.10±4.80	96.80±4.40	/	95.80±5.20	96.70±4.70	/
GS1	96.50±4.80	97.30±4.00	/	96.50±5.00	97.00±4.30	/
Cho’s	95.80±4.90	96.30±4.60	/	95.30±5.40	96.20±5.30	/
F-test	96.00±4.90	96.60±4.50	/	95.70±5.50	96.80±4.90	/

**Table 11:** The 5-fold CV classification accuracies of the KNN-classifier and SVM-classifier based on six gene selection methods on the SRBCT data.

Method	KNN			SVM		
	30	60	100	30	60	100
New method	98.27±0.91	98.87±0.38	99.06±0.53	97.71±0.81	99.73±0.50	99.82±0.43
Method in [28]	97.46±1.47	98.86±1.07	99.04±0.92	99.41±1.03	99.25±1.09	99.86±0.52
GS2	95.30±4.80	97.10±4.10	98.00±3.80	94.90±4.70	97.60±4.00	99.00±2.60
GS1	94.10±4.70	96.10±4.50	97.70±4.10	95.90±5.40	97.80±4.00	98.80±3.00
Cho’s	82.00±9.60	86.40±9.30	89.60±8.70	83.50±8.80	91.80±6.90	94.30±6.20
F-test	96.30±5.00	97.30±4.60	97.80±4.00	97.00±4.20	98.00±3.90	99.20±2.10

**Table 12:** The 5-fold CV classification accuracies of the KNN-classifier and SVM-classifier based on six gene selection methods on the LUNG data.

Method	KNN			SVM		
	30	60	100	30	60	100
New method	95.17±0.42	95.76±0.57	96.40±0.47	94.59±0.77	96.01±0.69	94.67±0.73
Method in [28]	92.13±0.57	94.82±0.50	94.91±0.57	90.82±1.00	97.66±0.47	96.47±0.71
GS2	88.40±5.30	91.60±4.10	92.80±3.70	85.80±6.10	91.30±3.50	93.10±3.30
GS1	89.00±4.60	91.90±4.10	93.70±3.40	87.10±5.10	92.20±3.80	93.80±3.10
Cho’s	84.30±5.30	89.70±4.40	92.40±3.80	80.30±6.50	89.40±4.40	92.40±3.50
F-test	87.30±4.90	88.20±4.40	91.80±4.40	85.20±5.50	90.10±4.20	93.00±3.60

**Table 13:** The 5-fold CV classification accuracies of the KNN-classifier and SVM-classifier based on two gene selection methods on the Colon data.

Method	KNN			SVM		
	30	60	100	30	60	100
New method	83.77±2.37	84.95±1.59	84.97±2.09	84.95±3.21	87.97±2.76	86.32±2.46
Method in [28]	75.95±2.01	80.90±2.01	81.03±2.01	84.05±3.43	80.18±3.46	79.56±3.73

Finally, to compare the KMeans-GCSI-MBPSO-ELM method with MIDClass [45], SGC (based on t-test (SGC-t) and based on WMW (SGC-W)) [44] and the method in [28], we conduct the experiments on the Brain cancer and Lymphoma data. To be consistent with [44], [45], the experiments are conducted with standard Leave-One-Out-Cross-Validation (LOOCV). The results of the KMeans-GCSI-MBPSO-ELM and the method in [28] is the average of 100 trials, and the results of the MIDClass and SGC methods are retrieved from [44], [45]. From the results shown in Table 14, it can be found that the KMeans-GCSI-MBPSO-ELM method outperform the method in [28], MIDClass and SGC methods.

**Table 14:** The LOOCV classification accuracies of five methods on the Brain cancer and Lymphoma data.

Data	LOOCV classification accuracy (%) (Gene number)				
	KMeans-GCSI-MBPSO-ELM	Method in [28]	MIDClass	SGC-t	SGC-W
Brain cancer	90.93 (6)	88.38 (5)	83 (239)	80 (1)	77 (1)
Lymphoma	93.79 (8)	91 (6)	69 (3)	76 (1)	71 (1)

### Discussion on the Selection of the Number of the Clusters

In the KMeans-GCSI-MBPSO-ELM method, the number of the clusters of the genes in the first-level initial gene pool (*N_c_*) is determined by trial and error where the number of the clusters is determined within the crossvalidation runs on the validation dataset. However, there are some methods available such as the Elbow method [51] or Silhouette scores [52] that could be used to determine the number of the clusters for K-means method. To show the influence of the selection of the *N_c_*, we use KMeans+Elbow-GCSI-MBPSO-ELM method to perform gene selection on six data. The only difference between the KMeans-GCSI-MBPSO-ELM and the KMeans+Elbow-GCSI-MBPSO-ELM method is the selection of the *N_c_*. In the KMeans+Elbow-GCSI-MBPSO-ELM method, the Elbow method [51] is used to determine the number of the clusters by evaluating the ratio of the between-group variance to the total variance (also known as an F-test). The comparison results between the KMeans-GCSI-MBPSO-ELM and the KMeans+Elbow-GCSI-MBPSO-ELM methods are provided in Table 15. The results in Table 15 are the average of 100 trials.

**Table 15:** The LOOCV and 5-fold CV classification accuracies of ELM based on two gene selection methods on the six data.

Data	KMeans-GCSI-MBPSO-ELM			KMeans+Elbow-GCSI-MBPSO-ELM		
	Classification accuracy±std		*N_c_*	Classification accuracy±std		*N_c_*
	LOOCV	5-fold CV		LOOCV	5-fold CV	
Leukemia	100.00±0.00	100.00±0.00	5	100.00±0.00	100.00±0.00	5
Colon	99.35±0.92	97.61±1.37	8	99.35±0.92	97.61±1.37	8
SRBCT	100.00±0.00	100.00±0.00	8	99.90±0.33	99.45±0.77	6
LUNG	98.14±0.33	97.10±0.63	10	97.33±0.66	95.67±0.74	6
Brain cancer	90.93±1.65	88.63±2.16	6	90.93±1.65	88.63±2.16	6
Lymphoma	93.79±2.07	86.97±2.44	7	93.79±2.07	86.97±2.44	7

From Table 15, the KMeans-GCSI-MBPSO-ELM and the KMeans+Elbow-GCSI-MBPSO-ELM methods select the same number of the clusters on some data including the Leukemia, Colon, Brain cancer and Lymphoma data, so ELM obtains the same classification accuracies with the genes selected by these two methods on these four data. The value of the *N_c_* in the KMeans-GCSI-MBPSO-ELM method is different from that of the KMeans+Elbow-GCSI-MBPSO-ELM method on both the SRBCT and LUNG data, and ELM obtains higher classification accuracies with the genes selected by the KMeans-GCSI-MBPSO-ELM method than with the one selected by the KMeans+Elbow-GCSI-MBPSO-ELM method on both the SRBCT and LUNG data. In summary, since the selection of the *N_c_* is determined by the crossvalidation accuracies obtained by ELM on the validation dataset in the KMeans-GCSI-MBPSO-ELM method, ELM tends to obtain higher classification accuracies on the full dataset with the genes selected by the KMeans-GCSI-MBPSO-ELM method than with the one selected by the KMeans+Elbow-GCSI-MBPSO-ELM method.

**Figure 8:**
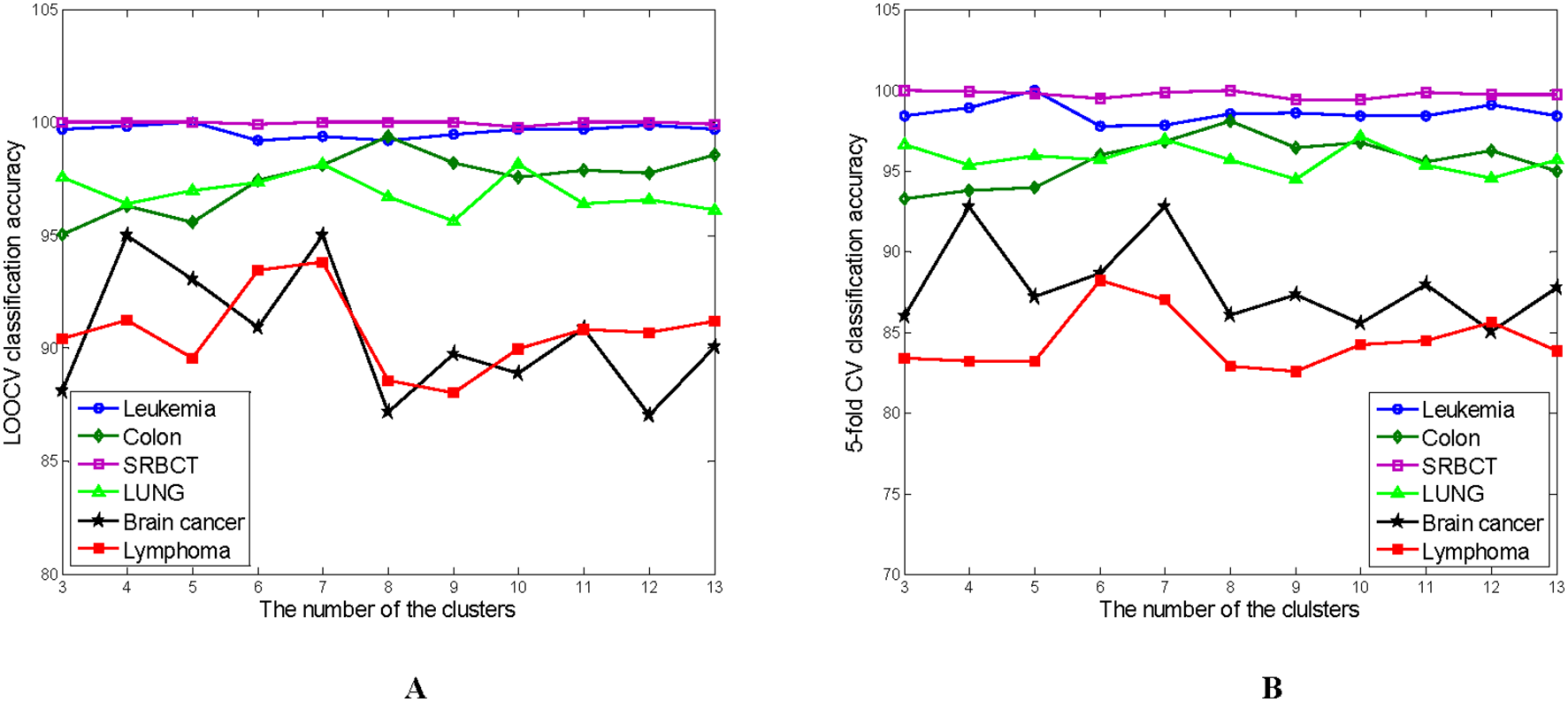
The number of the clusters (*N_c_*) versus the classification accuracy obtained by ELM with the genes selected by the KMeans-GCSI-MBPSO-ELM method on the six data.

Figure 8 shows the relationship between the number of the clusters (*N_c_*) and the classification accuracies on the full dataset obtained by ELM with the genes selected by the KMeans-GCSI-MBPSO-ELM method on the six data. The result in Figure 8 is the average of 100 trials. From Figure 8, the KMeans-GCSI-MBPSO-ELM method is less sensitive to the choice of the *N_c_* on the Leukemia and SRBCT data than on the other four data. The best *N_c_* for the KMeans-GCSI-MBPSO-ELM method is 5, 8, 8 and 10 on the Leukemia, Colon, SRBCT and LUNG data, respectively, which is same as the one determined by trial and error in the proposed method. The best *N_c_* is 4 and 7 on the Brain cancer data, and the best one is 6 on the Lymphoma data, while the *N_c_* is selected as 6 and 7 by trial and error in the KMeans-GCSI-MBPSO-ELM method on the Brain cancer and Lymphoma data, respectively. This difference lies in the fact that the *N_c_* in the KMeans-GCSI-MBPSO-ELM method is selected by trial and error within the crossvalidation runs on the validation dataset while the best *N_c_* obtained from Figure 8 is determined within the crossvalidation runs on the full dataset.

## Conclusions

In this study, the GCS information combined with K-means clustering method was used to reduce redundant genes, and then a modified BPSO encoding the GCS information was used to perform further gene selection. The new method could select the predictive gene subsets with comparatively high GCS values. Experiment results also shown that ELM, SVM and KNN classifiers obtained high prediction accuracy with the genes selected by the proposed method. Moreover, the proposed gene selection method has high reproducibility. Since the proposed method reduced the redundancy of genes only by removing the genes with low GCS values, it might filter out a few critical genes highly related to sample classification in some cases and thus lead into worse classification accuracy. Future work will include how to solve this problem in the proposed gene selection method as well as apply the new method to more complex microarray data.
